# Enhanced Biocide Treatments with D-amino Acid Mixtures against a Biofilm Consortium from a Water Cooling Tower

**DOI:** 10.3389/fmicb.2017.01538

**Published:** 2017-08-16

**Authors:** Ru Jia, Yingchao Li, Hussain H. Al-Mahamedh, Tingyue Gu

**Affiliations:** ^1^Department of Chemical and Biomolecular Engineering, Institute for Corrosion and Multiphase Technology, Ohio University, Athens OH, United States; ^2^Beijing Key Laboratory of Failure, Corrosion and Protection of Oil/Gas Facility Materials, Department of Materials Science and Engineering, China University of Petroleum – Beijing Beijing, China; ^3^Saudi Basic Industries Corporation Jubail, Saudi Arabia

**Keywords:** water cooling tower, biofilm, biocide, biocorrosion, D-amino acid, biofouling

## Abstract

Different species of microbes form mixed-culture biofilms in cooling water systems. They cause microbiologically influenced corrosion (MIC) and biofouling, leading to increased operational and maintenance costs. In this work, two D-amino acid mixtures were found to enhance two non-oxidizing biocides [tetrakis hydroxymethyl phosphonium sulfate (THPS) and NALCO 7330 (isothiazoline derivatives)] and one oxidizing biocide [bleach (NaClO)] against a biofilm consortium from a water cooling tower in lab tests. Fifty ppm (w/w) of an equimass mixture of D-methionine, D-leucine, D-tyrosine, D-tryptophan, D-serine, D-threonine, D-phenylalanine, and D-valine (D8) enhanced 15 ppm THPS and 15 ppm NALCO 7330 with similar efficacies achieved by the 30 ppm THPS alone treatment and the 30 ppm NALCO 7330 alone treatment, respectively in the single-batch 3-h biofilm removal test. A sequential treatment method was used to enhance bleach because D-amino acids react with bleach. After a 4-h biofilm removal test, the sequential treatment of 5 ppm bleach followed by 50 ppm D8 achieved extra 1-log reduction in sessile cell counts of acid producing bacteria, sulfate reducing bacteria, and general heterotrophic bacteria compared with the 5 ppm bleach alone treatment. The 10 ppm bleach alone treatment showed a similar efficacy with the sequential treatment of 5 ppm bleach followed by 50 ppm D8. The efficacy of D8 was found better than that of D4 (an equimass mixture of D-methionine, D-leucine, D-tyrosine, and D-tryptophan) in the enhancement of the three individual biocides against the biofilm consortium.

## Introduction

Large-scale cooling water systems are widely used to remove heat from industrial equipment using a heat exchanger in chemical manufacturing facilities, power plants and petroleum refineries (Wang et al., 2013). Natural water from a river, lake or sea can be used (Rajala et al., 2016). In some cooling water systems, the treated refinery wastewater was used (Liu F. et al., 2011). These water systems contain diverse species of microorganisms, organic matters and inorganic salts that enable microbial growth, leading to microbiologically influenced corrosion (MIC) and biofouling (Wang et al., 2012; Liu et al., 2013). Cooling tower systems typically have a water temperature between 25°C and 35°C that is ideal for microbes to grow (Liu Y. et al., 2011). Microbes attach to the surfaces to form biofilms by secreting extra polymeric substances (EPS) (Ghanbari et al., 2016). These biofilms can cause MIC and biofouling (Dobosz et al., 2015; Johnson et al., 2016; Ramírez et al., 2016; Wu et al., 2016).

Microbiologically influenced corrosion is a major problem in various industrial sectors, such as water utilities, oil and gas, and power generation (Qi et al., 2016; Xu et al., 2017; Jia et al., 2017c). MIC in recirculating cooling water systems causes deterioration of metallic surfaces and reduces the lifetime of the systems (Ilhan-Sungur and Çotuk, 2010). Biofouling on the other hand reduces the heat exchanging efficiency and hinders pipe flows (Trueba et al., 2013). Thus, these problematic biofilms reduce the reliability and increase the operating cost of the systems (Nebot et al., 2006).

Biocide dosing is a common approach to combat microorganisms in cooling water systems (Cloete et al., 1998). Chlorine is a widely used oxidizing biocide due to its low cost and high efficacy (Fagerlund et al., 2016; Gan et al., 2016). Other non-oxidizing biocides such as tetrakis hydroxymethyl phosphonium sulfate (THPS), glutaraldehyde, isothiazoline and quaternary ammonium compounds are also widely used in cooling towers (Miller and Koebel, 2006; Jia et al., 2017a). In the field, different microbes often live in biofilm communities (Marsh, 2005). Biofilms offer sessile cells protection from harmful environmental conditions and antimicrobial agents (Li et al., 2016). Therefore, a much higher concentration of biocide is required to treat biofilms than to treat planktonic cells due to the various defense mechanisms that biofilms use against antimicrobial agents (Mah and O’Toole, 2001). The high concentration raises operational and environmental problems. For example, chlorine at a high concentration causes equipment corrosion and is toxic to the environment after discharge (Rubio et al., 2015). In a recirculating cooling water system, continuous or cyclic biocide dosing is required because biofilms always bounce back (dos Santos et al., 2015). The repeated biocide dosing over time may cause dosage escalation due to biocide resistance. Thus, a more effective biocide treatment with reduced biocide dosages is highly desirable.

D-amino acids are naturally occurring chemicals. D-methionine (D-met), D-leucine (D-leu), D-tyrosine (D-tyr), and D-tryptophan (D-trp) were found to disperse *Bacillus subtilis, Pseudomonas aeruginosa*, and *Staphylococcus aureus* biofilms (Kolodkin-Gal et al., 2010). D-amino acids can enhance the efficacy of some existing biocides against corrosive biofilms. Lab tests showed that 1 ppm (w/w) D-tyr and 100 ppm D-met individually enhanced the efficacy of low concentrations of THPS and ADBAC (alkyldimethylbenzylammonium chloride) biocides, respectively in the mitigation of the *Desulfovibrio vulgaris* (a sulfate reducing bacterium) biofilm on carbon steel, achieving better efficacies than higher concentrations of THPS and ADBAC (Xu et al., 2012, 2014; Jia et al., 2017b). D-tyr at low concentrations (2–5 ppm) were found to enhance ciprofloxacin in the mitigation of anaerobic *P. aeruginosa* biofilms by achieving better efficacies than higher concentrations of ciprofloxacin (Jia et al., 2017d). It was suggested that a mixture of several D-amino acids was required to enhance THPS against field biofilm consortia because D-amino acids used individually showed limited effects (Li et al., 2016). In this work, two mixtures of D-amino acids containing equimass D-tyr, D-met, D-trp, and D-leu (labeled as D4) and equimass D-tyr, D-met, D-trp, D-leu, D-serine (D-ser), D-threonine (D-thr), D-phenylalanine (D-phe), and D-valine (D-val) (labeled as D8) were evaluated as biocide enhancers for bleach (active component: NaClO), THPS, and NALCO 7330 (active components: 5-chloro-2-methyl-4-isothiazolin-3-one and 2-methyl-4-isothiazolin-3-one) against a field biofilm consortium on C1018 carbon steel coupons retrieved from a water cooling tower.

## Materials and Methods

### Microbes and Chemicals

**FIGURE 1:**
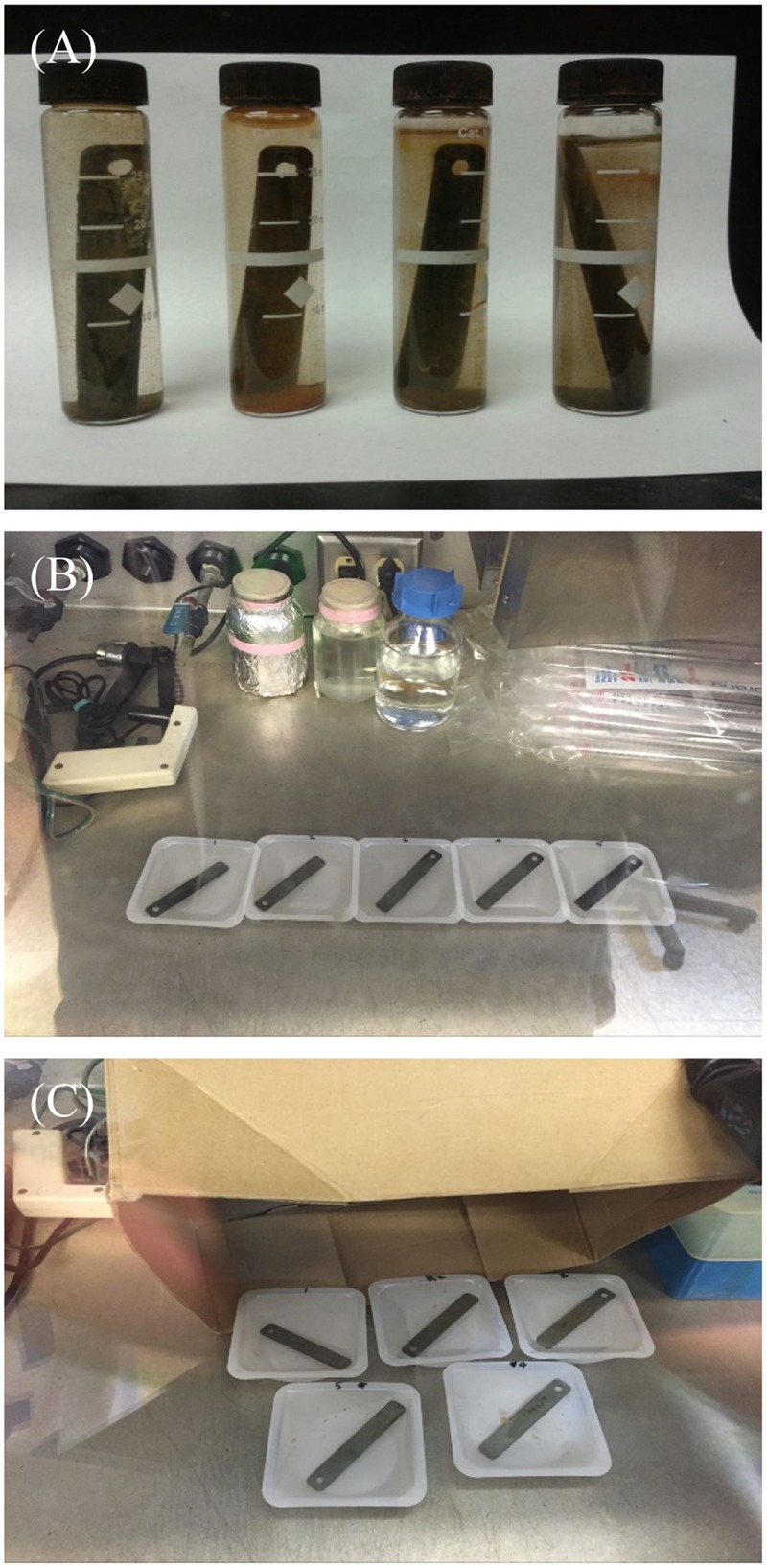
Experimental details: coupons with biofilms retrieved from a water cooling tower **(A)**, single-dose batch treatment (biofilms treated with different chemicals in weighing dishes) conducted in a biosafety cabinet **(B)**, and sequential treatment using bleach (biofilms treated with bleach followed by D-amino acids in weighing dishes) conducted in a biosafety cabinet with a cardboard box cover to avoid UV degradation **(C)**.

C1018 carbon steel coupons covered with biofilms were retrieved from a water cooling tower in a US chemical manufacturing facility after field exposure of 3 weeks (**Figure 1A**). The coupons were shipped overnight in capped vials to minimize deterioration of the biofilms. The biofilm morphology of the consortium on a coupon surface before the removal tests was observed using an SEM (scanning electron microscopy) (Model JSM-6390, JEOL, Tokyo, Japan). The detailed procedure of the coupon preparation for the observation under SEM was reported before (Jia et al., 2017b). The strip coupons (3″ × 0.5″ × 0.06″) were submerged in 30 ml vials with the fluid collected from the tower. NALCO 7330 was provided by the chemical manufacturing facility. D-amino acids were purchased from Sigma–Aldrich (St. Louis, MO, United States). Other chemicals used in this study were purchased either from Fisher Scientific (Pittsburgh, PA, United States) or Sigma–Aldrich (St. Louis, MO, United States). The biofilm treatment lab tests were conducted aerobically. Before each lab test, the PBS (phosphate buffered saline) buffer solution, tweezers, test tubes, and pipette tips were autoclaved at 121°C for 20 min. D-amino acid solutions were sterilized with a 0.22 μm Stericup filter (Millipore, Bedford, MA, United States). All experiments were conducted at least three times for accuracy.

### Enhanced Non-oxidizing Biocide Treatment against the Biofilm Consortium

Before the 3-h biofilm removal test, planktonic cells and the field fluid on the coupons were rinsed off using a pH 7.4 PBS buffer solution. Then, coupons were placed in weighing dishes with 100 ml of the PBS buffer with added treatment chemicals for 3 h in a biosafety cabinet at 25°C (**Figure 1B**). Biofilms were treated with non-oxidizing biocides (THPS and NALCO 7330 separately) with and without a D-amino acid mixture. The test matrix is shown in **Table 1**.

**Table 1:** Conditions for D-amino acid mixture enhancement of non-oxidizing biocides in the 3-h biofilm removal test.

Parameter	Condition
Biofilm	Consortium from a water cooling tower
Growth time	3 weeks
Solution	pH 7.4 PBS buffer solution
Treatment method	Non-oxidizing biocides, D-amino acid mixtures, non-oxidizing biocide + D-amino acid mixture
Treatment duration	3-h exposure to treatment chemicals in a weighing dish
Temperature	25°C
Coupon	C1018 carbon steel
Assay	Sessile cell counts, CLSM images

At the end of the 3-h treatment, the coupons were removed to enumerate sessile cells using the most probable number (MPN) method. MPN test kits were purchased from Biotechnology Solutions (Houston, TX, United States). Three liquid culture media, namely the modified Postgate’s B (MPB) medium for SRB (sulfate reducing bacteria), the standard bacterial nutrient broth for general heterotrophic bacteria (GHB), and the phenol red dextrose (PRD) medium for acid producing bacteria (APB), were used for MPN. After the biofilm removal test, coupons were rinsed with the PBS buffer solution to remove any loosely attached planktonic cells and treatment chemicals. The biofilm was scrapped off a coupon into a test tube with 10 ml PBS buffer using a small sterile brush. The scraped-off biofilm, the brush and the 10 ml PBS buffer were placed in the test tube. A vortex mixer was used for 30 s to suspend all the sessile cells evenly in the liquid before the liquid was used for MPN serial dilutions and incubation at 37°C. Each MPN enumeration was repeated twice for reproducibility. The commonly used *t*-test method was applied to obtain the *p*-value for statistical significance.

Live and dead sessile cells in biofilms on coupons were examined under confocal laser scanning microscopy (CLSM) (Model LSM 510, Carl Zeiss, Jena, Germany). The information on the staining procedure was described in a previous work (Jia et al., 2017b). The ImageJ software (National Institutes of Health, Bethesda, MD, United States) was used to quantify the live and dead sessile cells in CLSM images.

### Sequential Treatment Using Bleach and Different D-amino Acid Mixtures

The field coupons covered with biofilms (**Figure 1A**) were treated with bleach and D-amino acid mixtures. The operation was conducted in the biosafety cabinet with a cardboard box cover to prevent UV degradation due to exposure to light (**Figure 1C**). Since the bleach can react with D-amino acids, an abiotic chemical compatibility test was conducted first. Five ppm bleach (i.e., 5 ppm NaClO) was mixed with 10 ppm D-amino acid mixture in deionized water without inoculation for 3 h at 25°C. After that, the free chlorine concentration was measured using the “SenSafe Free Chlorine Water Check” test strips (Industrial Test Systems, Inc., Rock Hill, SC, United States).

In the 4-h biofilm removal test, bleach and a D-amino acid mixture were mixed to treat biofilms for 4 h. In addition to the single-dose batch treatment, a sequential treatment of bleach and a D-amino acid mixture was tried. During the sequential treatment, in the first 2 h, 5 ppm bleach was applied to treat a coupon. Then the coupon was retrieved and rinsed with the PBS buffer. It was then put into another weighing dish with 100 ml of the PBS buffer containing 50 ppm of a D-amino acid mixture for another 2 h. **Table 2** shows the test matrix. After the 4-h biofilm removal tests, the biofilms on coupons were examined under CLSM and the sessile cells on coupons were enumerated using the MPN test kits.

**Table 2:** Conditions for D-amino acid mixture enhancement of bleach in the 4-h biofilm removal test.

Parameter	Condition
Biofilm	Consortium from a water cooling tower
Growth time	3 weeks
Treatment method	Bleach, D-amino acid mixtures, bleach + D-amino acid mixtures
Treatment time	Control: 4 h without treatment D-amino acid mixtures alone: 2 h with 50 ppm D-amino acid mixtures + 2 h with no treatment Biocide alone: 2 h with 5 ppm bleach + 2 h with no treatment Sequential treatment: first 2 h with 5 ppm bleach followed by another 2 h with 50 ppm D-amino acid mixtures Biocide mixed with D-amino acids: 5 ppm bleach + 50 ppm D-amino acid mixture for 4 h Biocide alone: 2 h with 5 ppm bleach + 2 h with 5 ppm bleach
Temperature	25°C
Coupon	C1018 carbon steel
Assay	Sessile cell counts, CLSM images

## Results and Discussion

### Biofilm Consortium from the Water Cooling Tower

**FIGURE 2:**
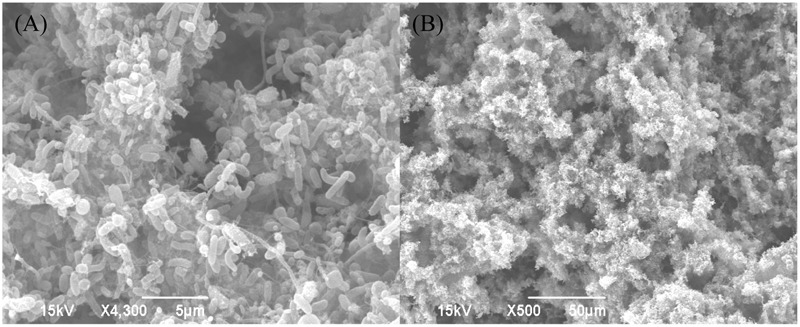
Scanning electron microscopy (SEM) images of the biofilm consortium on 3-week old C1018 coupons retrieved from the water cooling tower. Scale bars are 5 μm **(A)** and 50 μm **(B)**.

**Figure 2** shows the morphology of the biofilm consortium under SEM before testing. The image reveals that it is a mixed-culture consortium with sessile cells of different shapes. The phylogenetic identification of the biofilm consortium shown in **Table 3** was provided by Ecolyse, Inc. (College Station, TX, United States). The company used bacterial tag-encoded semi-conductor sequencing with 515F-GTGCCAGCMGCCGCGGTAA and 806R-GGACTACHVGGGTWTCTAAT as primers for the analysis. Samples were amplified for semi-conductor sequencing employing a forward primer and a reverse fusion primer. The amplification products were analyzed using eGels (Life Technologies, Grand Island, NY, United States). Sequencing was performed with the Ion Torrent PGM following manufacturer protocols (Life Technologies, Grand Island, NY, United States). The metagenomics data reveal that there were many species in the biofilm consortium. *Achromobacter marplatensis, Lacibacter cauensis*, and *Sphingobacterium* sp. are APB (Dees and Moss, 1978; Tamboli et al., 2010; Jin et al., 2013). *Algorimarina* sp. can grow as SRB (Kendall et al., 2006). *Arenimonas maotaiensis, Staphylococcus* sp., and *Terrimonas* sp. are GHB (Deurenberg and Stobberingh, 2008; Zhang et al., 2009; Gonzalez-Martinez et al., 2017). The trait of *Alkanindiges* sp., *Flavobacterium* sp., *Hydrogenophaga* sp., and *Novosphingobium* sp. is biodegradation, referring to bacterial utilization of substrates that cannot be used by most of the other bacteria (Bogan, 2003; Ortiz-Hernández et al., 2003; Gan et al., 2011; Chen et al., 2012).

**Table 3:** Metabolic assignments of microbial species (% of population).

Species	%
*Achromobacter marplatensis*	0.432
*Acidovorax* sp.	5.405
*Afipia* sp.	0.162
*Algorimarina* sp.	0.432
*Alkanindiges* sp.	1.189
*Arenimonas aquatica*	0.865
*Arenimonas maotaiensis*	6.378
*Arenimonas* sp.	0.486
*Bdellovibrio* sp.	0.378
*Cellvibrio* sp.	0.162
*Chlamydia* sp.	0.216
*Cytophaga* sp.	0.108
*Flavobacterium* sp.	8.541
*Gemmatimonas aurantiaca*	0.649
*Haliscomenobacter* sp.	0.649
*Hydrogenophaga* sp.	22.11
*Ideonella* sp.	1.514
*Lacibacter cauensis*	0.757
*Methylophilus* sp.	0.27
*Niabella* sp.	0.649
*Novosphingobium* sp.	1.081
*Ohtaekwangia* sp.	0.649
*Opitutus* sp.	0.541
*Pedobacter daechungensis*	0.595
*Rheinheimera* sp.	0.324
*Rhizobacteria* sp.	7.405
*Rhodobacter* sp.	0.865
*Sphingobacterium* sp.	0.324
*Sphingomonas* sp.	0.811
*Staphylococcus* sp.	0.27
*Terrimonas* sp.	3.027
*Thermomonas haemolytica*	0.378
*Thermomonas* sp.	3.081
*Unclassified*	24.81
*Variovorax* sp.	0.757
*Xanthomonas* sp.	3.73

### Removal Test Using Two Non-oxidizing Biocides and D-amino Acid Mixtures

**FIGURE 3:**
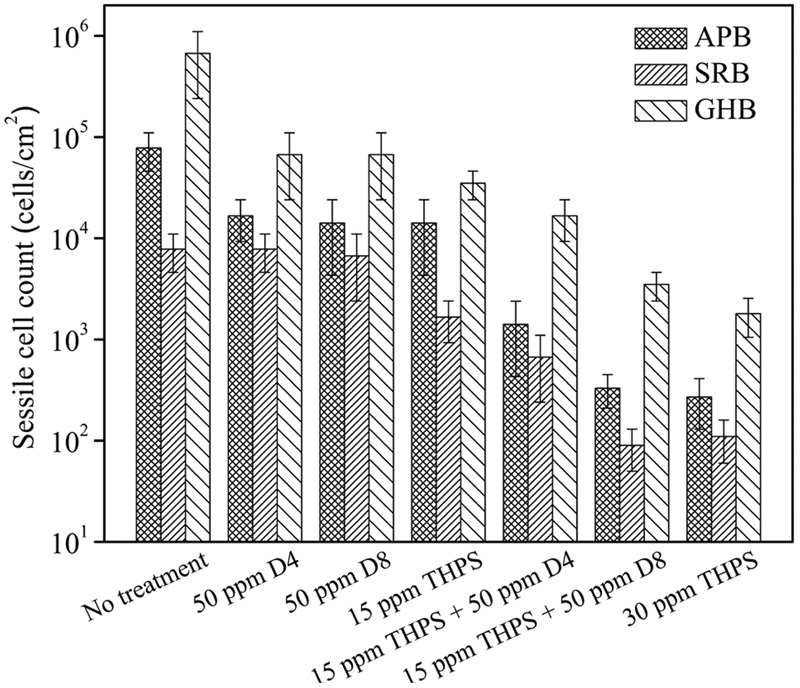
Sessile cell counts of the biofilm consortium after the 3-h biofilm removal test using THPS and D-amino acid mixtures. Error bars represent standard deviations from 4 independent samples.

**Figure 3** shows the sessile cell counts in the biofilm consortium treated with THPS and D-amino acid mixtures. After the 3-h biofilm removal test, the sessile cell counts on the no treatment control coupon were 7.8 × 10^4^ cells/cm^2^ APB, 7.8 × 10^3^ cells/cm^2^ SRB and 6.7 × 10^5^ cells/cm^2^ GHB, respectively. Fifty ppm D4 alone (*p* = 0.037) and D8 alone (*p* = 0.036) treatments both achieved 1-log GHB sessile cell reduction compared with the no treatment control. Compared with the no treatment control, no APB and SRB sessile cell reductions were observed with the 50 ppm D4 alone or 50 ppm D8 alone treatment. The 15 ppm THPS alone treatment only achieved 1.5-log GHB sessile cell reduction compared with the no treatment control (*p* = 0.032). The cocktail of 15 ppm THPS + 50 ppm D4 led to extra 1-log reduction of APB sessile cell count compared with the 15 ppm THPS alone treatment (*p* = 0.011). The treatment of 15 ppm THPS + 50 ppm D8 achieved extra 1.5-log reductions of APB sessile cell count (*p* = 0.008) and extra 1-log reduction of SRB (*p* = 0.005) and GHB (*p* = 0.004) in sessile cell counts, respectively compared with the 15 ppm THPS alone treatment. The outcome of the 15 ppm THPS + 50 ppm D8 treatment was similar to that of the 30 ppm THPS alone treatment. This means 50% reduction in THPS dosage was achieved. Results here indicate that D8 at the same concentration was more powerful than D4 in the enhancement of THPS against the biofilm consortium.

**FIGURE 4:**
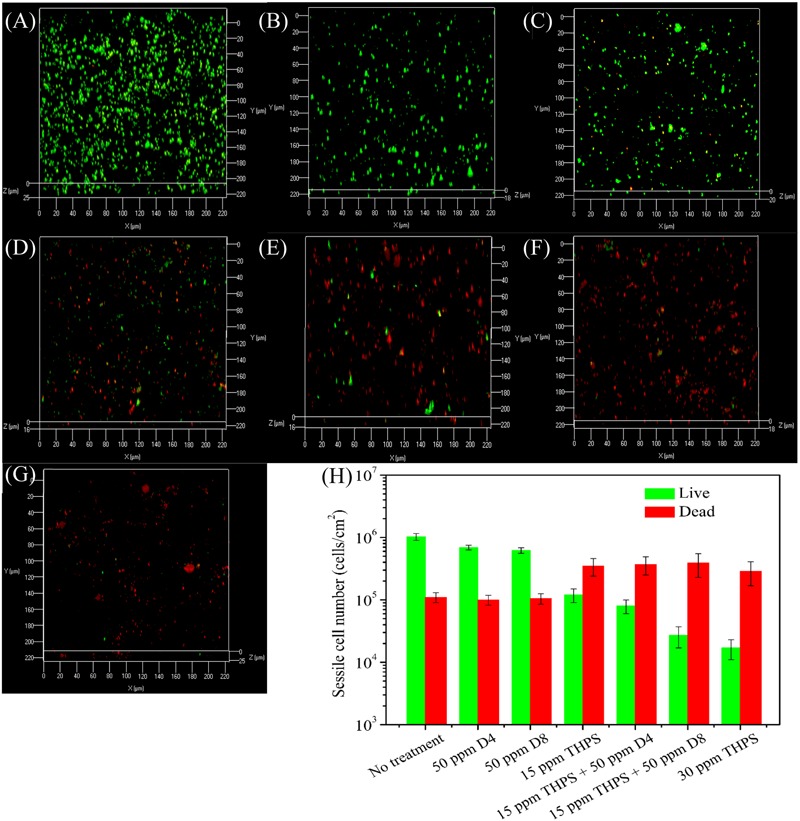
Confocal laser scanning microscopy (CLSM) images of biofilms after the 3-h biofilm removal test in the pH 7.4 PBS buffer containing: **(A)** no treatment (control), **(B)** 50 ppm D4-1, **(C)** 50 ppm D8-1, **(D)** 15 ppm THPS, **(E)** 15 ppm THPS + 50 ppm D4-1, **(F)** 15 ppm THPS + 50 ppm D8-1, and **(G)** 30 ppm THPS. The ImageJ software calculated numbers of live/dead sessile cells are shown in **(H)**. Error bars represent standard deviations from 4 independent samples.

**Figure 4** shows the CLSM biofilm images after different treatments using THPS. There were many live cells (green dots) on the no treatment control coupon (**Figure 4A**). Biofilm images after the 50 ppm D4 alone treatment, and the 50 ppm D8 alone treatment are shown in **Figures 4B,C**. The 15 ppm THPS alone treatment led to many dead cells (red dots) among live cells (**Figure 4D**). After the 15 ppm THPS + 50 ppm D4 treatment (**Figure 4E**), there were fewer live cells compared with the 15 ppm THPS alone treatment. After the 15 ppm THPS + 50 ppm D8 treatment (**Figure 4F**), there were much fewer live cells. Dead cells were abundant after the 30 ppm THPS alone treatment (**Figure 4G**). **Figure 4H** shows the numbers of live and dead sessile cells calculated from CLSM images in **Figure 4** using the ImageJ software. The results indicate that the mixture of D-amino acids alone had a very minor ability for the biofilm removal (*p* = 0.032). The cocktail of 15 ppm THPS + 50 ppm D8 achieved a better efficacy than the cocktail of 15 ppm THPS + 50 ppm D4 (*p* = 0.007). The efficacy of the cocktail of 15 ppm THPS + 50 ppm D8 was similar to that of the 30 ppm THPS alone treatment (*p* = 0.108). The CLSM data in **Figure 4** supported the MPN data in **Figure 3**.

**FIGURE 5:**
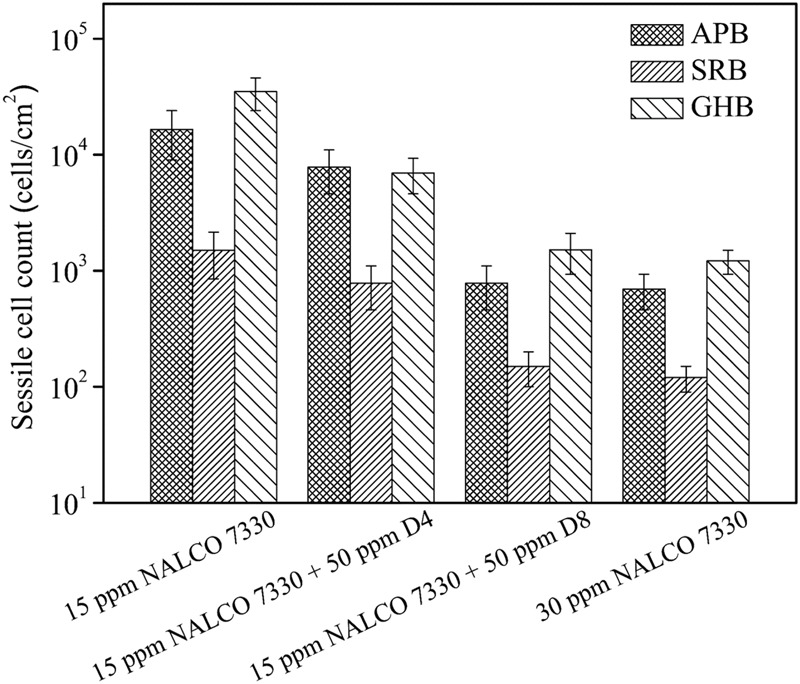
Sessile cell counts of the biofilm consortium after the 3-h biofilm removal test using NALCO 7330 and D-amino acid mixtures. Error bars represent standard deviations from 4 independent samples.

**FIGURE 6:**
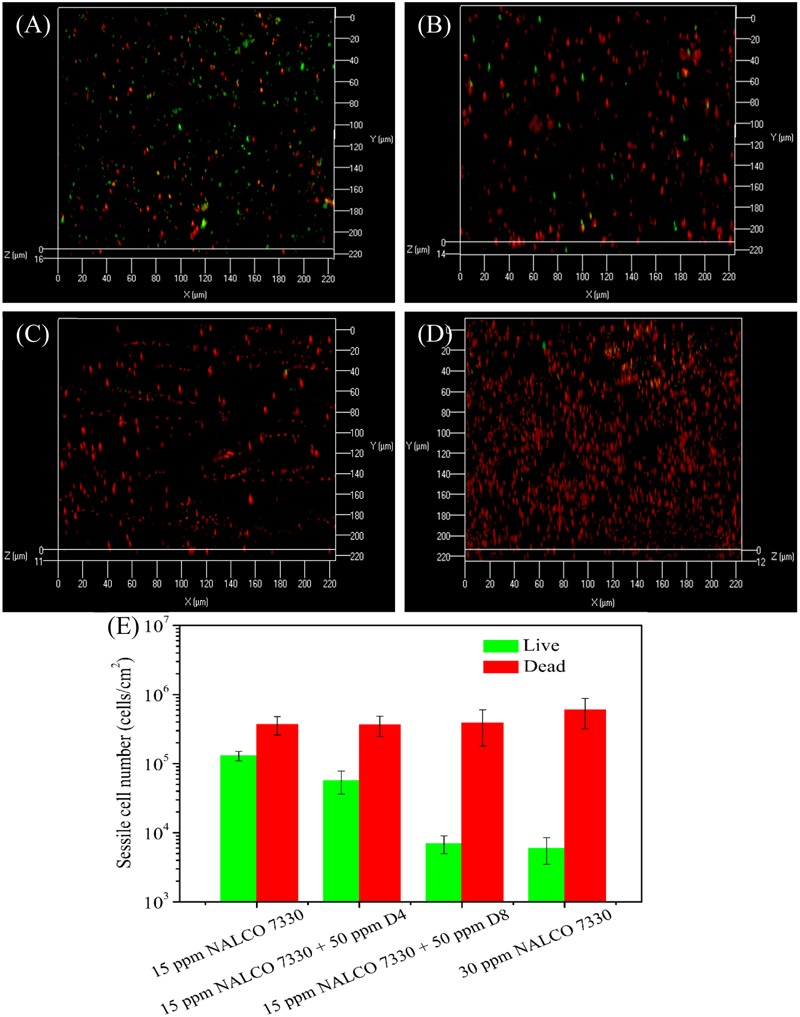
Confocal laser scanning microscopy images of biofilms after the 3-h biofilm removal test in the pH 7.4 PBS buffer containing: **(A)** 15 ppm NALCO 7330, **(B)** 15 ppm NALCO 7330 + 50 ppm D4-1, **(C)** 15 ppm NALCO 7330 + 50 ppm D8-1, and **(D)** 30 ppm NALCO 7330. The ImageJ software calculated numbers of live/dead sessile cells are shown in **(E)**. Error bars represent standard deviations from 4 independent samples.

In the 3-h biofilm removal test using NALCO 7330 (**Figure 5**), the 15 ppm NALCO 7330 alone treatment had a similar efficacy as the 15 ppm THPS alone treatment. The cocktail of 15 ppm NALCO 7330 + 50 ppm D4 led to extra 1-log reduction in GHB sessile cell count in comparison with the 15 ppm NALCO 7330 alone treatment (*p* = 0.006). The 15 ppm NALCO 7330 + 50 ppm D8 resulted in extra 1.5-log reductions of APB (*p* = 0.009) and GHB (*p* = 0.003) sessile cell counts and extra 1-log reduction of SRB (*p* = 0.008) sessile cell count in comparison with the 15 ppm NALCO 7330 alone treatment. The cocktail of 15 ppm NALCO 7330 + 50 ppm D8 achieved a similar efficacy to that achieved by the 30 ppm NALCO 7330 alone treatment. This means that 50 ppm D8 could reduce the biocide concentration by 50%. Results here demonstrate that the D-amino acid mixtures enhanced these non-oxidizing biocides at lower concentrations by achieving a similar efficacy as higher concentrations of non-oxidizing biocides. The CLSM images in **Figure 6** corroborated the sessile cell count results in **Figure 5**. With treatments of 15 ppm NALCO 7330 + 50 ppm D8 and 30 ppm NALCO 7330 alone, much fewer live cells were observed compared with those in the 15 ppm NALCO 7330 alone treatment. **Figure 6E** shows the numbers of live and dead sessile cells calculated from CLSM images in **Figure 6** using the ImageJ software. The cocktail of 15 ppm NALCO 7330 + 50 ppm D8 achieved a better efficacy than the cocktail of 15 ppm NALCO 7330 + 50 ppm D4 (*p* = 0.006). The efficacy for the cocktail of 15 ppm NALCO 7330 + 50 ppm D8 was similar to that for the 30 ppm NALCO 7330 alone treatment (*p* = 0.309). The enhanced non-oxidizing biocides with D-amino acid mixtures confirmed that a biocidal stress is necessary for D-amino acids to disperse the biofilm consortium (Li et al., 2016). Results also indicated that non-oxidizing biocides (THPS and NALCO 7330) and D-amino acids worked synergistically.

### Biofilm Removal Test Using Bleach and D-amino Acid Mixtures

**FIGURE 7:**
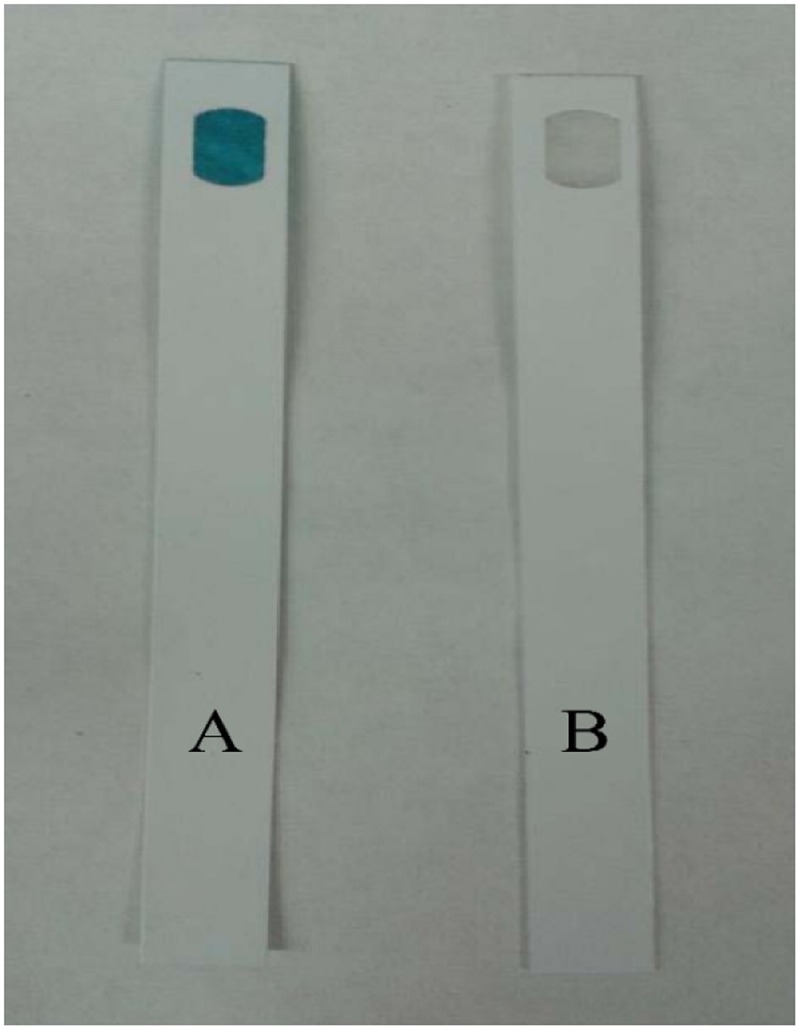
Bleach (containing 5 ppm NaClO) incubated with and without 10 ppm D-amino acid mixture in water at 25°C after 3 h: **(A)** bleach alone, and **(B)** bleach + 50 ppm D-amino acid mixture (D4 and D8 same outcome).

Chlorine is an oxidizing agent that can react with amino acids (Hureiki et al., 1994). In the compatibility test without inoculation, 50 ppm D-amino acid mixture (D4 or D8) were mixed with 5 ppm bleach (i.e., 5 ppm NaClO) in a dark environment for 3 h at 25°C. The free chlorine test strips can detect levels of ClO^-^ from 0 to 6 ppm, with a color range from white to dark blue. A white color means 0 ppm ClO^-^. **Figure 7** suggests that the D-amino acids depleted the ClO^-^ after 3 h. Thus, bleach and D-amino acids should not be dosed together.

**FIGURE 8:**
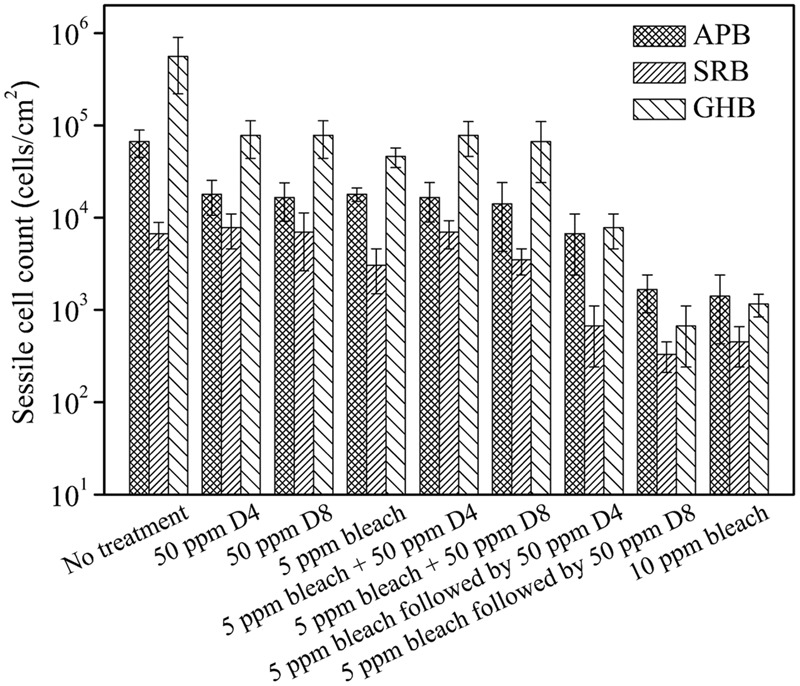
Sessile cell counts of the biofilm consortium after the 4-h biofilm removal test using bleach and D-amino acid mixtures. Error bars represent standard deviations from 4 independent samples.

At the end of the 4-h biofilm removal test, 50 ppm D4 alone (*p* = 0.035), 50 ppm D8 alone (*p* = 0.035), and 5 ppm bleach alone (*p* = 0.029) treatments (exposure to treatment chemicals for 2 h followed by exposure to PBS buffer for another 2 h) all achieved 1 log GHB sessile cell reduction in comparison with the no treatment control (**Figure 8**). When 5 ppm bleach and 50 ppm D4 or D8 were mixed to treat for 4 h zero-log reductions in APB, SRB, and GHB sessile cell counts were achieved in comparison with the treatment using 5 ppm bleach alone (lasting 2 h). The results confirmed that the chlorine reaction with D-amino acids rendered D-amino acids ineffective. Thus, a sequential treatment method was applied.

In the sequential treatment, the coupon was first immersed in 100 ml of the PBS buffer containing 5 ppm bleach. After 2 h, it was retrieved and placed into 100 ml fresh PBS buffer containing a 50 ppm mixture of D-amino acids. The sequential treatment of 5 ppm bleach followed by 50 ppm D4 achieved extra 1-log reductions of SRB (*p* = 0.031) and GHB (*p* = 0.002) sessile cell counts in comparison with the 5 ppm bleach alone treatment. The sequential treatment of 5 ppm bleach followed by 50 ppm D8 achieved extra 1-log reductions of APB (*p* = 0.001), SRB (*p* = 0.021), and GHB (*p* = 0.001) sessile cell counts in comparison with the 5 ppm bleach alone treatment. The 10 ppm bleach alone treatment showed a similar efficacy with the sequential treatment of 5 ppm bleach followed by 50 ppm D8. This means that 50 ppm D8 could reduce the bleach dose by 50%.

**FIGURE 9:**
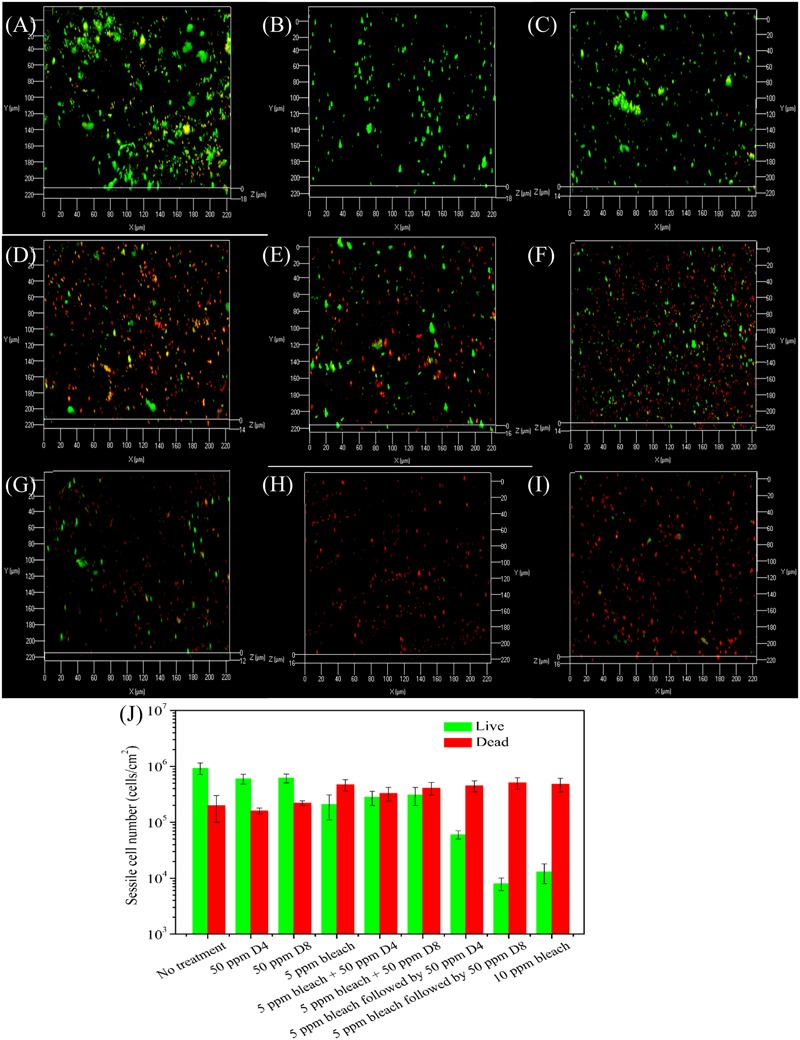
Confocal laser scanning microscopy images of biofilms after the 4-h biofilm removal test in the pH 7.4 PBS buffer containing: **(A)** no treatment for 4 h, **(B)** 50 ppm D4-1 alone for 2 h + no treatment for 2 h, **(C)** 50 ppm D8-1 alone for 2 h + no treatment for 2 h, **(D)** 5 ppm bleach alone for 2 h + no treatment for 2 h, **(E)** 5 ppm bleach + 50 ppm D4-1 for 4 h, **(F)** 5 ppm bleach + 50 ppm D8-1 for 4 h, **(G)** 5 ppm bleach for 2 h followed by 50 ppm D4-1 alone for 2 h, **(H)** 5 ppm bleach alone for 2 h followed by 50 ppm D8-1 alone for 2 h, and **(I)** 5 ppm bleach for 2 h + another 5 ppm bleach for 2 h (total 10 ppm bleach). The ImageJ software calculated numbers of live/dead sessile cells are shown in **(J)**. Error bars represent standard deviations from 4 independent samples.

The CLSM analysis in **Figure 9** supported the sessile cell count results in **Figure 8**. There were numerous live cells on the coupons from the no treatment control, the 50 ppm D4 alone treatment, and the 50 ppm D8 alone treatment as seen in **Figures 9A–C**. Many live sessile cells also appeared in the combination treatments using bleach + a D-amino acid mixture (**Figures 9E,F**). Fewer live cells appeared in sequential treatments (**Figures 9G,H**) in comparison with the bleach alone treatment (**Figure 9D**). The CLSM images also showed the efficacy of the sequential treatment of 5 ppm bleach followed by 50 ppm D8 (**Figure 9H**) was close to that of the 10 ppm bleach alone treatment (**Figure 9I**). **Figure 9J** shows the numbers of live and dead sessile cells calculated from CLSM images in **Figure 9**. The sequential treatments of 5 ppm bleach followed by 50 ppm D4 (*p* = 0.030) and 5 ppm bleach followed by 50 ppm D8 (*p* = 0.012) achieved a better efficacy than the 5 ppm bleach alone treatment. The efficacy for the sequential treatment of 5 ppm bleach followed by 50 ppm D8 was similar to that for the 10 ppm bleach alone treatment (*p* = 0.092).

**Figure 9** shows that 50 ppm D8 showed a better efficacy than 50 ppm D4. This was likely because different microbial species in the biofilm consortium responded to different D-amino acids. Thus, a mixture of more D-amino acids was better (Li et al., 2016). Several mechanisms have been suggested to explain why D-amino acids disperse bacterial biofilms. Researchers speculated that D-amino acids triggered biofilm disassembly because they replaced the D-alanine terminus in the peptidoglycan molecules that exist in all bacterial cell walls (Kolodkin-Gal et al., 2010). An addition of high concentration D-alanine to the culture medium was found to hinder the efficacy of D-methionine to enhance THPS against an SRB biofilm on carbon steel (Xu et al., 2014). D-amino acids were also suggested to influence the remodeling of bacteria cell walls (Lam et al., 2009; Cava et al., 2011). Leiman et al. (2013) found that D-amino acids inhibited the cell growth and expression of EPS.

## Conclusion

In this work, two different D-amino acid mixtures (D4 and D8) at 50 ppm enhanced two non-oxidizing biocides (THPS and NALCO 7330) against a field biofilm consortium from a water cooling tower. D8 was found to be more effective than D4 at the same concentration to enhance the biocides. Fifty ppm D8 enhanced 15 ppm THPS and 15 ppm NALCO 7330 by achieving similar efficacies as biocides at 30 ppm. A sequential treatment was tested for bleach due to its reactivity with D-amino acids. The sequential treatment of 5 ppm bleach followed by 50 ppm D8 achieved extra 1-log reductions in APB, SRB, and GHB sessile cell counts in comparison with the 5 ppm bleach alone treatment. The 10 ppm bleach alone treatment showed a similar efficacy with the sequential treatment of 5 ppm bleach followed by 50 ppm D8. This work demonstrated that 50 ppm D8 reduced the biocide dosages by 50% while achieving the same efficacies.

## Author Contributions

Conceived and designed the experiments: HA-M and TG. Performed the experiments: RJ and YL. Analyzed the data: RJ and YL. Wrote and polished the paper: RJ, YL, HA-M, and TG.

## Conflict of Interest Statement

The authors declare that the research was conducted in the absence of any commercial or financial relationships that could be construed as a potential conflict of interest.
